# Smoking, drinking, and depression: comorbidity in head and neck cancer patients undergoing radiotherapy

**DOI:** 10.1002/cam4.1497

**Published:** 2018-04-19

**Authors:** Kristen McCarter, Amanda L. Baker, Benjamin Britton, Luke Wolfenden, Chris Wratten, Judith Bauer, Sean A. Halpin, Gregory Carter, Alison K. Beck, Lucy Leigh, Christopher Oldmeadow

**Affiliations:** ^1^ School of Medicine and Public Health Faculty of Health and Medicine University of Newcastle PO BOX 833 Newcastle New South Wales 2300 Australia; ^2^ Department of Radiation Oncology Newcastle Mater Misericordiae Hospital Waratah New South Wales 2298 Australia; ^3^ Centre for Dietetics Research The University of Queensland St Lucia Queensland 4067 Australia; ^4^ School of Psychology Faculty of Science and IT University of Newcastle University Dr Callaghan New South Wales 2308 Australia; ^5^ Hunter Medical Research Institute and Faculty of Health and Medicine University of Newcastle LOT 1 Kookaburra Circuit New Lambton Heights New South Wales 2305 Australia

**Keywords:** Alcohol, comorbidity, depression, head neck cancer, smoking

## Abstract

We aimed to determine the prevalence and co‐occurrence of tobacco smoking, alcohol consumption, and depressive symptoms among a sample of head and neck cancer (HNC) patients undergoing radiotherapy. A total of 307 HNC patients participated in a multi‐site stepped‐wedge randomized controlled trial (RCT) evaluating the effectiveness of a dietitian‐delivered health behavior intervention in patients with HNC undergoing radiotherapy. During week one of radiotherapy patients completed measures of smoking, alcohol consumption, and level of depression. Approximately one‐fifth (21%) of patients had two or more co‐occurring problems: current smoking, hazardous alcohol use, and/or likely presence of a major depressive episode (MDE). Approximately one‐third (34%) of the sample were current smokers, one‐third (31%) were drinking hazardously and almost one‐fifth (19%) had likely cases of depression. Comorbidity of smoking, hazardous alcohol use, and MDE is high in HNC patients, and interventions need to address this cluster of cancer risk factors.

## Introduction

Tobacco smoking, alcohol consumption, and depressive symptoms are important determinants and highly prevalent factors in the onset, prognosis, and recovery from HNC. Continued smoking in cancer patients has been associated with several adverse outcomes including an increased risk for other smoking‐related diseases, second primary tumors, disease recurrence, poorer response to radiotherapy, decreased survival, and increased toxicity, and side effects from radiotherapy 1, 2, 3, 4. Continued alcohol intake at problematic levels has been associated with secondary cancers, decreased survival rates 5, and lower quality of life scores 3 in HNC patients. Depression in cancer patients is associated with increased morbidity and possibly increased mortality 6, 7. In HNC specifically, depressive symptoms have been associated with poorer quality of life scores 8 and found to be predictive of malnutrition during treatment 9.

Given the importance of these risks factors, a number of studies have described patterns of tobacco or alcohol use or depressive symptoms among HNC patients undergoing treatment. Prevalence estimates of these risk factors vary considerably. For example, evidence from observational and intervention studies report between one‐third and 75% of HNC patients continue to smoke after diagnosis 1, 10, 11. Between 37% and 54% of HNC patients continue to consume alcohol after diagnosis, with up to 16% continuing to drink at a hazardous level 10, 12. Estimates of the prevalence of depression in HNC patients range from 15% to 57% 8, 10, 13, 14. Variability in the reported consumption of alcohol and tobacco use among HNC patients, and the prevalence of depression in this group may be attributable to differences in the underlying prevalence rates in the population where the studies were conducted, period effects, or differences in measurement of tobacco 15 or alcohol use, 10, 12 or differences in diagnostic measurements for depression 16.

Of particular concern for both the immediate clinical outcomes of HNC patients, and their longer‐term health is the co‐occurrence of these risk factors. The presence of multiple health risk factors markedly increases the likelihood of adverse treatment outcomes. Smoking, alcohol misuse, and depressive symptoms tend to cluster and their relationship is complex 17. Despite the high rates of smoking, alcohol consumption, and depression reported in HNC patients and their effect on patient outcomes, there is little research investigating the rates of comorbidity of these factors in HNC patients. Duffy et al. 3, 10 conducted a cross‐sectional study, and a subsequent cohort study to examine the prevalence and associations between smoking, problem drinking, depressive symptoms, and quality of life among HNC patients recruited from Veterans Affairs hospitals in the USA, which included patients at varying stages of treatment. In the cross‐sectional study of 80 HNC patients, 76% scored positive for one or more of smoking, at‐risk alcohol intake and significant depressive symptoms. The follow‐up cohort study with a convenience sample of 973 HNC patients at varying stages of treatment reported similar results. However, the authors did not specify the prevalence of those patients with two or more of these issues.

Given the clinical and public health salience of tobacco smoking, alcohol consumption, and depressive symptoms among HNC patients, and the limitations of previous studies, a more comprehensive assessment of risk factors, and their co‐occurrence among HNC patients is required. Such information is important for health service planning and to ensure that care is provided to HNC patients that maximizes the likelihood of a positive long‐term prognosis. In particular, identification of those HNC patients who have co‐occurrence of smoking, alcohol consumption, and depressive symptoms before undergoing radiation treatment, may assist in considering interventions in addition to radiotherapy.

This is the first study to examine: smoking status; alcohol consumption; the severity of depressive symptoms; and their co‐occurrence assessed during the first week of radiotherapy. Our primary objectives were to:

1. Report the rates and severity of tobacco smoking, alcohol consumption, depressive symptom severity, and likelihood of major depressive episode (MDE); and
2. Describe the pattern of co‐occurrence of these factors

## Materials and Methods

### Procedures

In this cross‐sectional study, 307 patients participated in a multi‐site stepped‐wedge randomized controlled trial (RCT; Trial registration no. ACTRN12613000320752) and completed baseline assessments. The trial evaluated the effectiveness of training dietitians in an intervention based on psychological strategies (motivational interviewing and cognitive behavior therapy) in order to reduce malnutrition in patients with HNC undergoing radiotherapy 18. Eligible patients were approached with information about the study (by their radiation oncologist and/or an independent data manager) and written informed consent taken.

### Inclusion criteria

Patients eligible for inclusion in the trial met the following criteria:

- Aged 18 years or older.
- Pathologically confirmed diagnosis of HNC, involving the nasopharynx, oropharynx, oral cavity, larynx, or hypopharynx, requiring definitive or postoperative radiotherapy with curative intent.
- Receiving radiotherapy to a dose of at least 60 Gy with regional nodal irradiation including as a minimum ipsilateral nodal levels II–III.
- Available for follow‐up for at least 6 months post study initiation.
- Capacity to provide written informed consent.

### Exclusion criteria

- Inability to communicate in English.
- Presence of organic brain diseases (impairing ability to complete questionnaires satisfactorily).
- Likely insignificant oral or pharyngeal mucositis as a complication of radiotherapy treatment.

The study received approval from the Hunter New England Human Research Ethics Committee (HREC) of Hunter New England Health (HREC/12/HNE/108; HNEHREC: 12/04/18/4.06).

### Measures

Across five sites, during the first week of radiotherapy, an independent research officer administered assessment instruments. These included demographic information, patient clinical characteristics, measures of smoking and alcohol consumption, and related features (level of nicotine dependence, intentions to change smoking or alcohol consumption), and level of depression. The research officer also conducted chart reviews to extract cancer diagnosis, staging, and treatment data.

### Demographic characteristics

Demographic information included age (years), gender (male/female), marital status, Aboriginal and Torres Strait Islander (ATSI) status, education, accommodation, and employment status.

### Clinical characteristics

Clinical information included tumor site, tumor stage, proposed radiotherapy dose, proposed chemotherapy, surgery, and feeding tube status (prophylactic percutaneous endoscopic gastrostomy; PEG or nasogastric tube; NGT).

### Smoking

Patients were asked about their smoking behavior (ever smoked, current smoker, most recent cigarette, number of cigarettes within the last 24 h, current nicotine replacement therapy; NRT use).

Expired carbon monoxide (CO) provided biochemical verification of smoking status. The Micro 11 Smokerlyser assessed breath levels of CO for all patients. A cut‐off of ≥4 CO parts per million (PPM) was used to classify abstinence from smoking, as has been suggested to increase specificity in determining smoking abstinence, particularly for those patient groups that might be more inclined to misrepresent their smoking status as has been found in HNC patients 19, 20, 21, 22, 23, 24 ^,^.

Nicotine dependence was measured via the Fagerstrom Test for Nicotine Dependence (FTND) – a six‐item, reliable, and valid self‐report questionnaire designed to assess the strength of nicotine dependence 25. Item scores are summed to produce a total score, with higher scores indicating higher levels of nicotine dependence (0–2 = very low; 3–4 = low; 5 = medium; 6–7 = high; 8–10 = very high dependence).

As a descriptive measure of chronicity and severity of smoking and alcohol consumption, intention to change was assessed using an adapted version of the measure developed by Etter and Sutton 26. For smoking, participants were asked to indicate the statement that best reflected their current plan to quit smoking:

*I am not thinking about quitting in the near future, I intend to quit in the next 6 months, I intend to quit in the next 30 days, I have quit in the last 6 months*,* I have quit for 6 months or more,* or *Not applicable – Never smoked*.

### Alcohol consumption

The Alcohol Use Disorders Identification Test (AUDIT) 27 is a ten‐item self‐report measure developed by WHO to identify harmful patterns of alcohol use over the preceding 12 months. Items are summed to produce a total score, with scores >=8 indicating harmful or hazardous alcohol use, as well as possible alcohol dependence.

The AUDIT‐Consumption 27 consists of the first three items of the AUDIT and provides an index of alcohol use. It was employed to detect changes in quantity and/or type of alcohol consumed more recent to the start of treatment and referred to alcohol use in the preceding 2‐months. A score of ≥4 in men and a score ≥3 or more in women is considered positive for identifying hazardous drinking.

For intention to change assessment, participants were asked to indicate the statement that best reflected their current plan to cut down on drinking; *I am not thinking about cutting down in the near future, I intend to cut down in the next 6 months, I intend to cut down in the next 30 days, I have cut down in the last 6 months,* or *I have cut down for 6 months or more*. This was measured even in those who reported never having a drink in the last two months, as the statements include options for having cut down.

### Depression

The PHQ‐9 28 is a self‐administered nine‐item questionnaire that can either be scored continuously to assess depressive symptoms (depressive severity), or scored categorically to assess the likely presence of a major depressive episode (MDE). Participants are asked to rate (on a scale of 0–3) the frequency of various MDE criteria over the previous 2 weeks. A cut‐off score of ≥8 has been suggested for identifying MDE in cancer patients 29, and the severity of the depression can be rated as 0–4 = minimal; 5–9 = mild; 10–14 = moderate; 15–19 = moderately severe; 20–27 = severe.

### Statistical analysis

Descriptive statistics (means, SD, and frequencies) were conducted on all demographic and health variables, smoking, nicotine dependence, alcohol consumption, and depressive symptoms. Crosstab analyses were conducted to examine the co‐occurrence of current smoking (≥4 CO PPM), hazardous alcohol use (AUDIT‐C score ≥3 for women, ≥4 for men), and likely presence of MDE (PHQ‐9 score ≥8).

## Results

### Patient characteristics

The sample is described in Table 1. The mean age was 58 (*SD* 10) and most were male. Just over half (56%) had cancer of the oropharynx and most had stage IV (65%) cancer. All patients were scheduled to undergo radiotherapy; about a third had surgery prior to radiotherapy. Almost a quarter (23%) had a PEG feeding tube prior to starting radiotherapy and only 2% had a NGT.

**Table 1:** Patient characteristics of head and neck cancer patients at week one of radiotherapy (*N *=* *307)

Variable	*N*/Mean	%/SD
Age (years)	58	10.4
Sex		
Male	244	80%
Female	63	21%
Country		
Australia	198	65%
UK & Ireland	38	12%
Other	71	23%
Primary language		
English	285	93%
Other	22	7%
ATSI		
Yes	6	2%
No	300	98%
Marital status		
Married	156	51%
De‐facto/common law couples	37	12%
Widowed	12	4%
Separated/divorced	57	18%
Single, never married	40	13%
Other	5	2%
Education level		
4 years of high school or less	112	36%
6 years of high school	155	50%
University/Vocational College	146	48%
Other	1	<1%
Accommodation (past year)		
Private residence (own home, private rental)	297	97%
Partially supported living (Department of housing, independent unit in retirement village/nursing home)	9	3%
Other	1	<1%
Employment (past year)		
No job	19	6%
Full time	152	50%
Part time/casual	31	10%
Housework/stay at home parent	7	2%
Studying	2	1%
Retired/volunteer	84	27%
Other	12	4%
Tumor site		
Nasopharynx	23	8%
Oropharynx	171	56%
Oral Cavity	66	22%
Larynx	29	9%
Hypopharynx	11	4%
Unknown Primary	7	2%
Tumor stage		
I	12	4%
II	39	13%
III	57	19%
IV	199	65%
Radiotherapy	307	100%
Surgery prior to radiotherapy	97	32%
Concurrent chemotherapy	247	81%
Prophylactic PEG	71	23%
Prophylactic NGT	7	2%
Hospital site		
Site 1	23	8%
Site 2	100	33%
Site 3	83	27%
Site 4	101	33%

### Smoking, alcohol, and depression

Baseline smoking, alcohol consumption, and depressive symptoms are presented in Table 2.

**Table 2:** Smoking, alcohol consumption, and depressive symptoms at baseline

Variable	*N* (%)
Smoking	
Current smoker (self‐report) (*n *=* *304)	40 (13%)
Number of cigarettes within last 24 h (*n *=* *38)	
0–9	28 (74%)
10–20	9 (24%)
21–30	1 (3%)
Ever smoked (*n *=* *305)	232 (76%)
Currently using NRT (*n *=* *232)	18 (9%)
Most recent cigarette (*n *=* *230)	
<24 h	38 (17%)
<2 weeks	11 (5%)
<1 month	11 (5%)
<6 months	46 (20%)
<1 year	9 (4%)
<5 years	16 (7%)
>5 years	99 (43%)
Nicotine dependence; FTND (patients who had smoked in the last month) (*n *=* *53)	
Very low	19 (36%)
Low	19 (36%)
Medium	6 (11%)
High	9 (17%)
Very high	0
CO confirmed current smokers (*n *=* *280)	
CO PPM ≥4	94 (34%)
Intentions to change (smoking) (*n *=* *295)	
I am not thinking about quitting in the near future	15 (5%)
I intend to quit in the next 6 months	15 (5%)
I intend to quit in the next 30 days	16 (5%)
I have quit in the last 6 months	67 (23%)
I have quit for 6 months or more	113 (38%)
Not applicable – Never smoked	71 (24%)
Alcohol consumption	
AUDIT (past year)	
Frequency of use (how often do you have a drink containing alcohol?) (*n *=* *303)	
Never	46 (15%)
Monthly or less	56 (19%)
2 to 4 times a month	34 (11%)
2 to 3 times a week	57 (19%)
4 or more times a week	109 (36%)
Typical consumption (alcohol drinks on a typical day when drinking?) (*n *=* *257)	
1–2	129 (50%)
3–4	66 (26%)
5–6	43 (17%)
7–9	5 (2%)
10 or more	14 (5%)
Frequency of 6 or more standard drinks on one occasion (*n *=* *257)	
Never	111 (43%)
Less than monthly	54 (21%)
Monthly	40 (16%)
Weekly	30 (12%)
Daily or almost daily	22 (9%)
Harmful/hazardous use (AUDIT ≥8) (*n *=* *294)	77 (30%)
AUDIT‐C (past 2 months)	
Frequency of use (how often do you have a drink containing alcohol?) (*n *=* *306)	
Never	114 (37%)
Monthly or less	47 (15%)
2 to 4 times a month	38 (12%)
2 to 3 times a week	47 (15%)
4 or more times a week	60 (20%)
Typical consumption (alcohol drinks on a typical day when drinking?) *(n *=* *192)	
1–2	119 (62%)
3–4	39 (20%)
5–6	23 (12%)
7–9	2 (1%)
10 or more	9 (5%)
Frequency of 6 or more standard drinks on one occasion (*n *=* *192)	
Never	133 (70%)
Less than monthly	18 (9%)
Monthly	14 (7%)
Weekly	14 (7%)
Daily or almost daily	13 (7%)
Hazardous drinking (AUDIT‐C ≥ 3 for women, ≥4 for men) (*n *=* *306)	94 (31%)
Intentions to change (alcohol use) (*n *=* *301)	
I am not thinking about cutting down in the near future	140 (47%)
I intend to cut down in the next 6 months	7 (2%)
I intend to cut down in the next 30 days	14 (5%)
I have cut down in the last 6 months	105 (35%)
I have cut down for 6 months or more	35 (12%)
Depressive symptoms (PHQ‐9) (*n *=* *303)	
Minimal	197 (65%)
Mild	70 (23%)
Moderate	22 (7%)
Moderately severe	11 (4%)
Severe	3 (1%)
Likely presence of MDE (PHQ‐9 score ≥8)	58 (19%)

### Comorbidity

**Figure 1:**
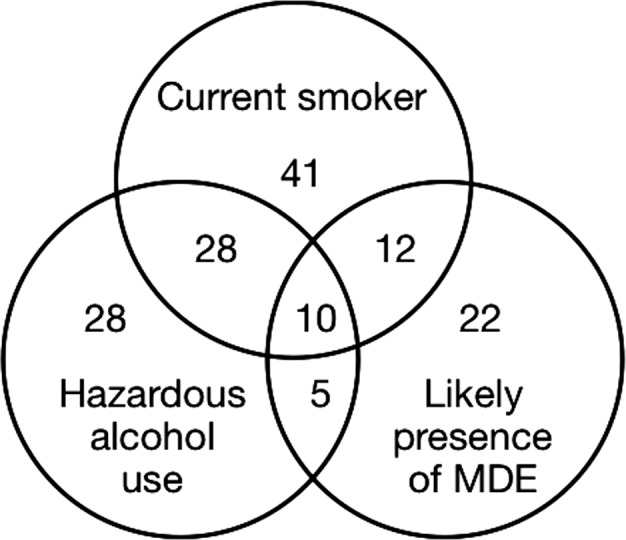
Comorbidity at baseline.

Of 276 patients with complete data for all three outcomes, 21% scored positive for two or more of the following problems: current smoking (≥4 CO PPM), hazardous alcohol use (AUDIT‐C score ≥3 for women, ≥4 for men), and likely presence of MDE (PHQ‐9 score ≥8) (Fig. 1). For those patients who had ever smoked and reported reducing their alcohol intake (from 4 or more times per week in the 12 months before baseline to less than that in the 2 months before baseline), 32% (*n *=* *13/41) also reported quitting smoking recently (i.e., their last cigarette was between 2 weeks and 6 months prior to baseline).

## Discussion

### Comorbidity

This is the first study to examine: smoking status; alcohol consumption; the severity of depressive symptoms; and their co‐occurrence assessed during the first week of radiotherapy. Approximately one‐fifth of the sample (*n *=* *59/276; 21%) scored positive for two or more problems; smoking, hazardous alcohol consumption, and probable depression. Interestingly, of patients who reported i) having smoked in their lifetime; and ii) reducing their alcohol intake prior to baseline, approximately one‐third reported quitting smoking relatively recently. This is in line with previous research that suggests smoking cessation can enhance sobriety from alcohol, as opposed to impede alcohol abstinence 30.

In an oncology setting, HNC patients may feel overwhelmed by recent diagnosis, treatment schedules, and side effects. Health professionals’ focus may be primarily on treating the malignancy and resources and time are limited. In such circumstances, it would be valuable to treat comorbid problems together rather than separately. There is little research investigating effective interventions for such comorbidity in this population. The co‐occurrence of reduced alcohol intake and smoking in our participants prior to baseline, demonstrates the potential for concurrent reductions in smoking and alcohol use in the HNC population. For those HNC patients who continue to smoke, drink alcohol at hazardous levels or experience depressive symptoms during treatment and particularly those with co‐occurrence of these issues, a multicomponent, intensive treatment may be beneficial 10.

### Smoking

Consistent with previous research, approximately one‐third (34%) of the sample were current smokers (CO PPM) at the beginning of radiotherapy 1, 10. Coupled with the potential for those who had recently quit to relapse over the course of treatment, assessment of smoking status, and the development of cessation interventions in this group warrants attention. A recent review 31 found that very few smoking cessation trials have been conducted with the HNC population but that a multicomponent approach (i.e., pharmacotherapy and evidence‐based psychosocial therapies) may be beneficial, also addressing co‐occurring risk factors.

Self‐reported smoking status was also much lower than reported in previous studies with HNC patients 1, 10. Given the research that suggests patients may minimise their smoking status, particularly in smoking‐related cancers, it may be that some patients misrepresent their smoking status 22. There is evidence that for some cancer patients, particularly those with smoking‐related cancers such as HNC, diagnosis is sufficient to produce abstinence 11. However, there is also a considerable rate of relapse for HNC patients who quit smoking; as high as between 13 and 90% depending on follow‐up period 21, 32, 33. Given the evidence that demonstrates the negative effects of tobacco use on treatment outcomes and survival, smoking status should be measured and biochemically confirmed at diagnosis, throughout treatment and at follow‐up in this population 33, with a view to offering assistance with smoking cessation interventions.

### Alcohol consumption

Compared to smoking, less research has been conducted on alcohol consumption in HNC patients and the results of our study help to characterize this health behavior in this cancer population. The rate of alcohol consumption (last 12 months 85%; last 2 months 63%) in our sample was comparable to that of current drinkers (75%) in a sample (*n *=* *107) of newly diagnosed HNC patients 34. Further, about one‐third of our sample scored positive for hazardous drinking, relative to the past 2 months (AUDIT‐C). This finding combined with those who were found to be at risk of alcohol‐related disorders (30%; AUDIT; past 12 months) is similar to rates described in previous studies 3, 12, 35. Given the association between problem drinking and secondary cancers, decreased survival rates and poorer quality of life 3, 5, this degree of problem drinking in HNC patients is concerning.

It has been suggested that the high rate of continued drinking in this population may be in part explained by lack of patient awareness of the association between alcohol and HNC 12. Indeed, almost half of our sample endorsed “I am not thinking about cutting down (alcohol use) in the near future.” Health care personnel across numerous specialties have reported that they do not deem discussing alcohol acceptable 36. However, physicians involved in the treatment of HNC patients are well placed to provide information about the hazards of continued drinking and studies of primary care patients have demonstrated that most are open to advice from physicians about their alcohol use 37. This opportunity for intervention is especially important in HNC patients where alcohol consumption in combination with smoking is responsible for the majority of these cancers 38 and continued use increases the risk of a secondary cancer 5.

Despite the high rate of current drinking at baseline, a proportion of patients had cut down on drinking four or more times per week from 36% in the last 12 months to 20% in the last 2 months. More patients were also drinking in the lower range of drinking on a typical day in the past 2 months as compared to the past 12 months. It may be that as for smoking, the symptoms or diagnosis of cancer is sufficient to change alcohol use for some, whilst others who continue to drink at harmful levels despite a cancer diagnosis need additional support to cut down.

### Depression

Almost one‐fifth (19%) of our patients were identified as having likely cases of depression using the PHQ‐9. This is consistent with the lower range of rates reported in the HNC literature 8, 10, 13, 14. Identifying the prevalence of depression in HNC patients is complicated by the use of varying screening and diagnostic tools, unclear reporting of depression diagnoses versus depressive symptoms and time of measurement (e.g., pre or post cancer treatment). However, even conservative estimates of depressive symptoms and likely cases of depression in this cancer population at the pretreatment stage warrants attention. The importance of screening for depression and offering referral for psychosocial support has been highlighted in the numerous evidence‐based cancer guidelines that recommend this delivery of care.

### Limitations

A limitation of the study is that although a valid self‐report tool was used to measure the likelihood of meeting criteria for a major depressive disorder, this was not confirmed by a diagnostic assessment and may have resulted in an overestimation of patients with depression. The patients in our sample were undergoing treatment with curative intent. Consequently, our findings are limited to this population.

## Conclusions

The occurrence of smoking, alcohol consumption, and depressive symptoms was considerable. For a sizeable group of patients, these problems were co‐occurring. Screening and assessment of these behaviors and conditions should be conducted prior to treatment in order to provide intervention for those who continue to smoke or for recent quitters, consume alcohol, or experience depression. Additional support may be necessary for a subgroup with comorbid issues. Treating smoking, hazardous alcohol use, and/or depressive symptoms is likely to be associated with improved treatment outcomes and greater survival in HNC patients.

## Conflict of Interest

This work was supported by the National Health and Medical Research Council (APP1021018; 2011/3654). This funder had no role in the study design; in the collection, analysis, and interpretation of data; in the writing of the report; and in the decision to submit the article for publication. The authors report no proprietary or commercial interest in any product mentioned or concept discussed in this article.
